# Endogenous Melanin and Hydrogen‐Based Specific Activated Theranostics Nanoagents: A Novel Multi‐Treatment Paradigm for Rheumatoid Arthritis

**DOI:** 10.1002/advs.202401046

**Published:** 2024-04-26

**Authors:** Lin Chen, Mingxin Zhao, Weiwei Kang, Lujie Yu, Chongqing Zhang, Shutong Wu, Xiaorui Song, Keqi Zhao, Pengmin Liu, Qin Liu, Rong Dai, Ziliang Zheng, Ruiping Zhang

**Affiliations:** ^1^ Department of Radiology Fifth Hospital of Shanxi Medical University (Shanxi Provincial People's Hospital) Taiyuan 030000 China; ^2^ Academy of Medical Sciences Shanxi Medical University Taiyuan 030001 China; ^3^ Medical Imaging Department Shanxi Province Cancer Hospital (Shanxi Hospital Affiliated to Cancer Hospital Chinese Academy of Medical Sciences/Cancer Hospital Affiliated to Shanxi Medical University) Taiyuan 030001 China; ^4^ Shanxi Bethune Hospital, Shanxi Academy of Medical Sciences Tongji Shanxi Hospital, Third Hospital of Shanxi Medical University Taiyuan 030032 China

**Keywords:** hypoxic‐responsive, multi‐treatment, rheumatoid arthritis, second near‐infrared fluorescence imaging, theranostics

## Abstract

Rheumatoid arthritis (RA) is a systemic autoimmune disorder characterized by excessive proliferation of rheumatoid arthritis synovial fibroblasts (RASFs) and accumulation of inflammatory cytokines. Exploring the suppression of RASFs and modulation of the RA microenvironment is considered a comprehensive strategy for RA. In this work, specifically activated nanoagents (MAHI NGs) based on the hypoxic and weakly acidic RA microenvironment are developed to achieve a second near‐infrared fluorescence (NIR‐II FL)/photoacoustic (PA) dual‐model imaging‐guided multi‐treatment. Due to optimal size, the MAHI NGs passively accumulate in the diseased joint region and undergo rapid responsive degradation, precisely releasing functionalized components: endogenous melanin‐nanoparticles (MNPs), hydrogen gas (H_2_), and indocyanine green (ICG). The released MNPs play a crucial role in ablating RASFs within the RA microenvironment through photothermal therapy (PTT) guided by accurate PA imaging. However, the regional hyperthermia generated by PTT may exacerbate reactive oxygen species (ROS) production and inflammatory response following cell lysis. Remarkably, under the acidic microenvironment, the controlled release of H_2_ exhibits precise synergistic antioxidant and anti‐inflammatory effects with MNPs. Moreover, the ICG, the second near‐infrared dye currently approved for clinical use, possesses excellent NIR‐II FL imaging properties that facilitate the diagnosis of deep tissue diseases and provide the right time‐point for PTT.

## Graphical abstract

In this work, the novel hypoxia‐responsive nanoagents (MAHI NGs) are developed to release the functional components: endogenous melanin‐nanoparticles (MNPs), hydrogen gas (H_2_), and indocyanine green (ICG), which achieved an accurate NIR‐II FL/PA imaging‐guided multi‐treatment paradigm for rheumatoid arthritis (RA) management.

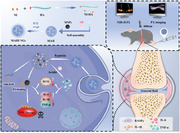

## 1 Introduction

Rheumatoid arthritis (RA) is deemed as a systemic, inflammatory, clinical autoimmune syndrome featuring excessive reproduction of rheumatoid arthritis synovial fibroblasts (RASFs), inflammatory/immune cell infiltration, ultimately cartilage degradation, bone tissue erosions, and joint destruction.^[^ ^1^, ^2^, ^3^ ^]^ Unfortunately, owing to its obscure pathogenesis, RA remains a significant challenge in clinical diagnosis and treatment. For one thing, the clinical imaging modalities frequently used for RA, such as radiography (RX) and magnetic resonance (MR) imaging, consistently demonstrate low sensitivity and accuracy in tracing early RA.^[^ ^4^, ^5^ ^]^ Thus, there is an urgent need for a molecular imaging technique with superior spatiotemporal resolution, higher signal‐to‐noise ratio, and low phototoxicity to facilitate precise diagnosis of RA.^[^ ^6^ ^]^ For another, the existing treatments for RA, such as disease‐modifying antirheumatic drugs (DMARDs), biological agents (tocilizumab), and glucocorticoids (GCs), commonly exhibit several shortcomings such as single therapeutic function and unclear deliverability, which limits therapeutic efficiency.^[^ ^7^, ^8^ ^]^ The more severe deficiency is that these traditional medicines exhibit limited efficacy and imprecise targeting for RASFs proliferation in RA. The RASFs are considered to generate oxidative stress and release a broad range of inflammatory cytokines in pathological research, including interleukin‐6 (IL‐6), interleukin‐1β (IL‐1β), and tumor necrosis factor‐α (TNF‐α).^[^ ^9^, ^10^, ^11^ ^]^ Fortunately, photothermal therapy (PTT), an innovative non‐invasive therapeutic intervention with high specificity and exceptional therapeutic efficiency, is utilized to induce effective cell lysis at a relatively high temperature (>42 °C).^[^ ^12^, ^13^ ^]^ The PTT agents exhibit the capacity to convert light into heat energy via nonradiative decay.^[^ ^14^, ^15^ ^]^ Regional hyperthermia could inhibit DNA synthesis, impair some repair processes, and change the cellular membrane fluidity, thereby resulting in the ablation of RASFs in the end. Owing to the robust NIR absorption characteristics, endogenous melanin nanoparticles (MNPs) demonstrate both excellent photoacoustic (PA) imaging and PTT capabilities, which can be used for PA imaging‐guided PTT to optimize the time‐point of the irradiation to maximize the effect of PTT.^[^ ^16^ ^]^ However, the irreversible damage of RASFs caused by PTT triggers oxidative stress, which stimulates the accumulation of reactive oxygen species (ROS), maturation and release of inflammatory factors, and a series of inflammatory reactions.^[^ ^17^ ^]^ The PTT not only fails to regulate the oxidative imbalance accompanied by RA but also exacerbates ROS generation and inflammatory response, resulting in the so‐called “vicious cycle” within the RA synovial microenvironment.^[^ ^18^ ^]^ Therefore, the combination of effective ROS clearance, inhibition of inflammatory factors expression, and elimination of RASFs could be an attractive strategy for RA treatment.

As traditional anti‐inflammatory strategies, aspirin and other nonsteroidal anti‐inflammatory drugs (NSAIDs) still exist several limitations including inefficiency and toxic side effects due to biological barriers during treatment, which demonstrates the imperative necessity for novel and intelligent anti‐inflammatory agents with a high level of biosecurity and superior diffusion properties.^[^ ^19^ ^]^ Endogenous MNPs, possessing good biocompatibility, exhibit dual functionality in ROS scavenging and suppressing inflammatory responses by inhibiting the expression of inflammatory mediators and cytokines, which greatly mitigates the deleterious effects induced by PTT and effectively harmonizes the RA microenvironment.^[^ ^20^ ^]^ Gas therapy has garnered significant research attention due to its remarkable anti‐inflammatory and antibacterial properties. The inherent capacity of gas molecules to freely traverse biological membranes without specific transporter proteins facilitates effective, safe, and non‐toxic therapies through synergistic coordination or induction of target cell apoptosis. Among the numerous therapeutic gases (CO, NO, H_2_S, etc.), hydrogen gas (H_2_), as a reductive agent with good biocompatibility, can rapidly capture and eliminate the free ROS and inflammatory factors. Therefore, the precise collaboration between H_2_ and MNPs exhibits precise synergistic antioxidant and anti‐inflammatory properties, significantly mitigating the detrimental effects resulting from PTT and orchestrating the RA microenvironment harmoniously and effectively.^[^ ^21^, ^22^, ^23^, ^24^ ^]^ However, a key challenge associated with H_2_ therapy is the difficulty of accumulating adequate H_2_ at local disease sites. Owing to the high diffusivity and low solubility, the accumulation of H_2_ is significantly hindered, which limits the efficiency of H_2_ therapy, particularly when targeting specific regions affected by diseases like RA. Therefore, achieving site‐specific delivery and controlled release of H_2_ emerges as a critical pursuit in the realm of H_2_ therapy.^[^ ^25^ ^]^

**Scheme 1:**
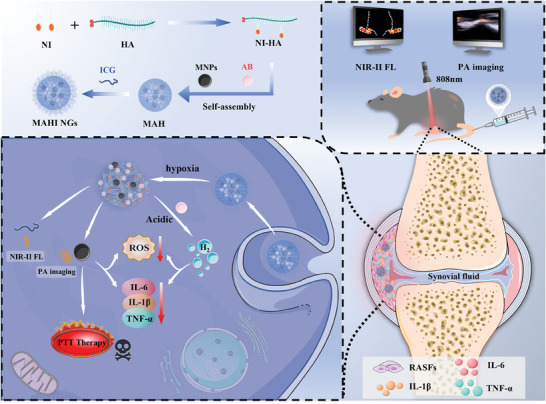
The schematic diagram for the synthesis and application of MAHI NGs in RA multi‐treatment.

The high metabolic state of the RASFs and immune cells, along with inefficient vascular oxygen perfusion, lead to a highly hypoxic and weakly acidic microenvironment in the RA lesion.^[^ ^26^, ^27^, ^28^ ^]^ Developing drugs for H_2_ release under endogenous stimulus‐responsiveness is one of effective strategies to achieve H_2_‐controlled release. Hence, the hypoxic properties of the RA microenvironment have been designed to construct hypoxia‐responsive nanoagents that precisely release functionalizing components, enabling dual‐modal imaging‐guided multi‐treatment (PTT, antioxidative, and anti‐inflammatory). In this study, we developed hypoxia‐responsive nanoagents (MAHI NGs) based on MNPs/AB/2‐nitroimidazole‐hyaluronic‐acid (NI‐HA) modified by indocyanine green (ICG) on the surface for achieving the second near‐infrared (NIR‐II, 1000–1700 nm) fluorescence (FL)/PA imaging‐guided multi‐treatment in RA (**Scheme** **1**). Owing to good stability and superior biocompatibility, the MAHI NGs achieved efficient accumulation in the RA area of collagen‐induced arthritis (CIA) mice via extravasation through leaky vasculature and subsequent inflammatory cell‐mediated sequestration (ELVIS) effect. Once inside the RA region, 2‐nitroimidazole (2‐NI) could convert into hydrophilic 2‐aminoimidazole (2‐AI) activated by the hypoxia microenvironment. This transformation triggers a rapid degradation of the MAHI NGs, facilitating the controlled release of functionalized components (MNPs, AB, and ICG). The released MNPs, acting as excellent PA imaging and PTT agents, play a critical role in ablating the RASFs within the RA microenvironment via PTT guided by precise PA imaging. However, the regional hyperthermia resulting from PTT can trigger oxidative stress and inflammatory responses following cell lysis. Fortunately, the collaboration between MNPs and H_2_ controllably released in the acidic condition exhibits precise synergistic antioxidant and anti‐inflammatory effects. In addition, ICG serves as a widely recognized clinical NIR dye sanctioned by the Food and Drug Administration (FDA) in the U.S., which facilitates the diagnosis of deep tissue diseases and provides the right time‐point for PTT owing to the outstanding NIR‐II FL imaging property.^[^ ^29^, ^30^, ^31^ ^]^ Consequently, based on endogenous MNPs and H_2_‐specific hypoxia microenvironment activation, the novel theranostics nanoagents could be an efficient NIR‐II FL/PA imaging‐guided multi‐treatment paradigm for RA.

## 2 Results and Discussion

**Figure 1:**
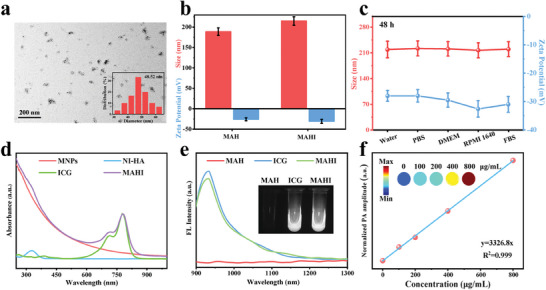
Characterization and optical properties of MAHI NGs. a) TEM image of MAHI, where the size distribution is shown in the inset. b) The hydrodynamic size and zeta potentials of MAH and MAHI. c) The DLS and zeta potentials of MAHI dispersed in different solutions (Water, PBS, DMEM, RPMI 1640, and FBS) at 37 °C within 48 h. d) The UV‐vis spectra of MNPs, NI‐HA, ICG, and MAHI aqueous dispersion. e) The NIR‐II FL spectrum of MAH, ICG, and MAHI. Inset: NIR‐II FL images of MAH, ICG, and MAHI under 808 nm excitation. f) The corresponding quantitative concentration‐dependent photoacoustic signal values of MAHI.

The amphiphilic NI‐HA conjugate was prepared by grafting 2‐NI to the HA backbone. Next, the hydrogen donor AB and endogenous MNPs were encapsulated into the NI‐HA by a self‐assembly method as well as ICG modified on the surface of MAH (MNPs/AB/NI‐HA). As shown in **Figure** **1**a, the transmission electron microscope (TEM) images of MAHI NGs displayed multinuclear‐coated particles with a size of ≈48.52 nm. Subsequently, the hydrodynamic diameter and zeta potential of nanoagents with and without modified ICG were compared in Figure 1b. It was vital to emphasize that the hydrodynamic size increased from 195 to 215 nm and the zeta potential changed from −26 ± 3.6 to −31 ± 4.9 mV, indicating the successful modification of ICG on the MAH surface. Interestingly, the dimensions measured by dynamic light scattering (DLS) were larger than those recorded by TEM, which might account for the existence of hydrated layers. The stability of MAHI is a prerequisite for their further application. No significant changes in average hydrodynamic size and zeta potential of MAHI NGs (≈219 nm and −30 mV) were observed over 48 h in various solutions, suggesting excellent stability in physiological media (Figure 1c). It facilitated the targeting of MAHI NGs to inflamed tissues after the intravenous administration and accumulation, which was conducive to maximizing the treatment effect.

As depicted in Figure 1d, the UV‐vis spectroscopy of MAHI NGs exhibited two specific absorption peaks at 330 nm (NI) and 805 nm (ICG), respectively, illustrating the successful self‐assembly of the NI‐HA carrier and modification with ICG.^[^ ^32^ ^]^ Inspiringly, the broad absorbance from UV‐vis to the NIR range of MNPs in MAHI NGs would be beneficial for PA imaging and PTT. Notably, the NIR‐II FL spectrum and intensity of MAHI NGs aligned closely with free ICG molecules at the same ICG concentration, indicating that the loading procedure did not significantly alter the NIR‐II FL emission property of ICG, which was beneficial for its application as an imaging agent in vivo (Figure 1e). Activated by the excellent absorption of MAHI NGs, we further investigated the potential of nanoagents as a PA imaging contrast agent. As shown in Figure 1f, the PA intensity of MAHI NGs linearly depended on the sample concentrations under 808 nm laser irradiation, confirming the excellent PA imaging capability in vivo. This above analysis indicated that the MAHI NGs have tremendous potential to be utilized for NIR‐II FL/PA dual‐modal imaging.

**Figure 2:**
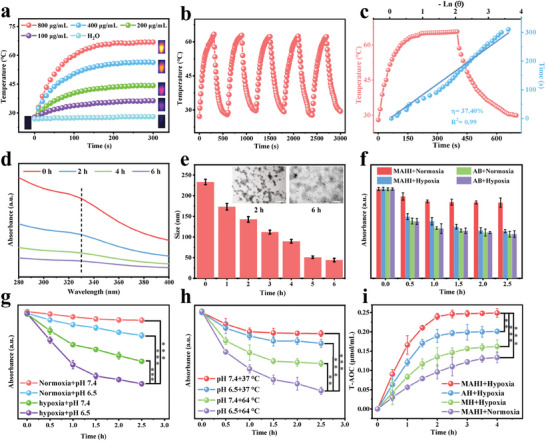
Photothermal effect and hypoxic‐responsive behavior of MAHI NGs in vitro. a) Photothermal effect of MAHI NGs with different concentrations upon 5 min of irradiation (808 nm, 1.0 W cm^−2^). b) Photothermal stability of MAHI NGs (800 µg mL^−1^) under five heating–cooling cycles (808 nm, 1.0 W cm^−2^). c) Photothermal efficiency evaluation of MAHI NGs (800 µg mL^−1^) under irradiation of an 808 nm laser (1.0 W cm^−2)^ which was turned off after irradiation for 300 s. In vitro degradation behavior of the MAHI NGs under hypoxic conditions for different time‐points via d) UV‐vis spectra e) DLS distribution and TEM images. f–h) Real‐time monitoring of MAHI NGs hydrogen release under various treatments utilizing MB as a probe for active hydrogen. i) Antioxidant capability evaluation in different conditions. Trolox was used as a control standard. Mean ± SD, n = 4, ^*^ *p* < 0.05, ^**^ *p* < 0.01, ^***^ *p* < 0.001.

Encouraged by the excellent NIR absorption performance, we recorded the temperature change at various concentrations of the MAHI NGs solution under 808 nm laser irradiation within 5 min. As depicted in **Figure** **2**a, the temperature rises of MAHI NGs were found to be irradiation duration and concentration‐dependent, demonstrating that MAHI NGs can efficiently and rapidly convert the 808 nm laser light into thermal energy. Strikingly, the temperature of MAHI was raised to 67 °C with a concentration of 800 µg mL^−1^. Furthermore, the MAHI NGs solution was irradiated with an 808 nm laser (1.0 W cm^−2^) for 5 min and subsequently cooled the solution to room temperature (Figure 2b). It was worth noting that the changes are almost the same during the five laser on‐off cycles, indicating stable thermal behavior of the MAHI NGs. Remarkably, the efficiency of photothermal conversion was an important parameter in judging the quality of photothermal agents. According to the cooling curves and the linear time date and −ln*θ*, the photothermal conversion efficiency (η) of MAHI NGs was up to 37.40%, which could be promised to be a new photothermal agent with great potential in bioapplication (Figure 2c).

Based on the hypoxic‐responsive property of NI structures, we evaluated the degradation behavior of MAHI NGs in the RA microenvironment of simulation. As seen in Figure 2d, The typical absorption peak of 2‐nitroimidazole (at 330 nm) declined dramatically over time and vanished completely after 6 h, which was attributed to the transformation of the hydrophobic molecule 2‐nitroimidazole into the hydrophilic molecule 2‐aminoimidazole within MAHI NGs under hypoxic conditions.^[^ ^33^, ^34^ ^]^ The changes in hydrodynamic size and TEM images of the nanoagents at various degrading time points were recorded to further verify the hypoxia‐responsive capacity of MAHI NGs (Figure 2e, inset). The MAHI NGs exhibited a substantial decrease in size and morphological transformation within 6 h, proving that the small‐sized core components were released, and the structure of MAHI NGs was compromised. Such a transition from hydrophobicity to hydrophilicity would favorably decompose MAHI NGs to release the functionalized components (AB and MNPs).

The H_2_ used in this study was provided via a low‐releasing donor (AB). Utilizing the inherent capability of H_2_ to progressively reduce methylene blue (MB), we assessed the H_2_ generation efficiency of MAHI NGs by monitoring the standard absorption peak of MB under diverse pH values and temperature conditions, in both normoxic and hypoxic environments.^[^ ^35^ ^]^ Under hypoxic conditions, the H_2_ release of MAHI NGs was more sensitive than in the normoxic condition, confirming that the AB‐protective capability of the NI‐HA shell allowed for the H_2_‐releasing process to be activated on demand (Figure 2f). The H_2_ release of MAHI NGs was accelerated significantly under the hypoxic + pH 6.5 condition, indicating that MAHI NGs could be stimulated to release H_2_ in the RA microenvironment (Figure 2g). Benefiting from the superior photothermal properties of MAHI NGs, photothermally promoted H_2_ generation at different pH values has also been discovered in hypoxic conditions. As illustrated in Figure 2h, the results showed a significant increase in H_2_ release under pH 6.5 + 64 °C, indicating that the photothermal effect could significantly accelerate the rate of H_2_ release. In addition, the total antioxidant capability (T‐AOC) of MAHI NGs was investigated by applying 2,2′‐azino‐bis (3‐ethylbenzthiazoline‐6‐sulfonic acid) (ABTS). With MNPs/NI‐HA (MH) and AB/NI‐HA (AH) in the hypoxia environment as the control groups, MAHI NGs exhibited the best T‐AOC over time in the hypoxia environment (Figure 2i). Consequently, all the above results amply indicated that MAHI NGs could realize the functionalized components (MNPs and AB) release controllably and rapidly under the stimulus of the RA microenvironment (hypoxic condition) to achieve the PTT and synergistic antioxidant effect.

**Figure 3:**
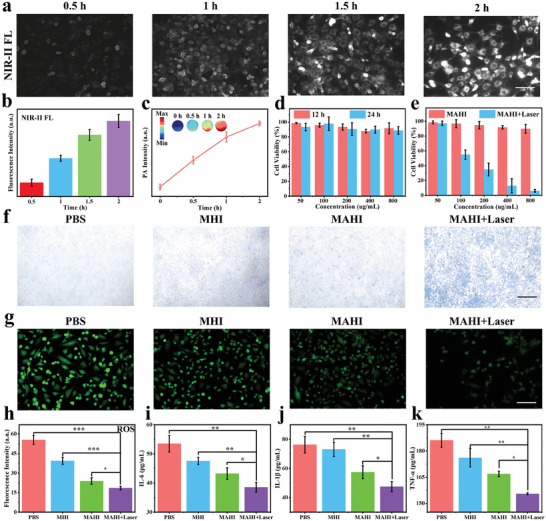
Cellular uptake and evaluation of multi‐therapeutic effects at the cellular level. a,b) NIR‐II FL, and c) optoacoustic imaging of the RASFs after incubation with MAHI NGs at different time points. Scale bar: 50 µm. d) Relative cell viability of HUVECs exposed to various concentrations of MAHI NGs for 12 h or 24 h. e) Relative viabilities of RASFs treated with different concentrations of MAHI NGs with/without laser irradiation (808 nm, 1 W cm^−2^). f) Trypan blue staining photos of RASFs treated with PBS, MHI, MAHI, and MAHI+Laser Scale bar: 100 µm. g) Fluorescence images stained with DCFH‐DA probe after different treatments (PBS, MHI, MAHI, MAHI+Laser) and h) fluorescence intensity of correspondence. Scale bar: 50 µm. i–k) The change of intracellular inflammatory cytokines (IL‐6, IL‐1β, and TNF‐*α*) in RASFs after various treatments. Mean ± SD, n = 3, ^*^ *p* < 0.05, ^**^ *p* < 0.01, ^***^ *p* < 0.001.

The hyperproliferation and infiltration of RASFs played a critical role in the progress of RA, which could stimulate angiogenesis, secrete inflammatory cytokines, and invade surrounding articular cartilage. Before verifying the therapeutic effect of MAHI on RASFs, we utilize NIR‐II FL and PA imaging to trace the endocytosis process at various incubation times of 0.5, 1, 1.5, and 2 h. With time prolongation, the NIR‐II FL signal and the corresponding quantitative analysis of intensity gradually enhanced and reached the strongest intensity in 2 h, revealing the excellent cellular endocytosis capabilities of MAHI NGs (**Figure** **3**a,b). Subsequently, we further evaluated the cellular uptake capacity by PA imaging. The PA intensity progressively improved with the prolongation of internalization time within 2 h (Figure 3c). Before evaluating the corresponding treatment effect in vitro, the cytotoxicity of both the MAHI NGs and free small molecules was assessed in human umbilical vein endothelial cells (HUVECs) by the standard Cell Counting Kit‐8 (CCK‐8) assays. No apparent cytotoxicity was observed at all tested concentrations after 12 or 24 h of incubation with MAHI NGs, which suggested the good biocompatibility and biosafety of the nanoagents (Figure 3d). Similarly, the MNPs and ICG revealed negligible cytotoxicity at various concentrations by CCK‐8 assay, demonstrating the biocompatibility after the release of MAHI NGs under the RA microenvironment (Figure S1, Supporting Information). Additionally, the CCK‐8 assays were also utilized to judge the effect of PTT on the RASFs under different conditions. After exposure to NIR laser irradiation, the cell cytotoxicity increased quickly in a dose‐dependent manner, which showed the minimum cell viability at the MAHI concentration of 800 µg mL^−1^ (Figure 3e). We also analyzed the RASFs with different treatments by trypan blue staining, and the cell mortality of the MAHI + Laser group was significantly higher than that of the PBS, MHI, and MAHI groups. These results indicated the significant PTT ability of MAHI NGs on the RASFs (Figure 3f).

In the RA microenvironment, the excessive generation of ROS causes aberrant redox homeostasis, and removing ROS may inhibit the pathological progress of RA.^[^ ^36^ ^]^ To further investigate the ROS scavenging ability of MAHI within the intricate biological surroundings, we utilized a 2′,7′‐dichlorofluorescein diacetate (DCFH‐DA) fluorescence probe to conduct the ROS cleaning capability in vitro, which could produce green fluorescence after oxidization by ROS.^[^ ^37^ ^]^ As shown in Figure 3g, obvious green fluorescence signals were exhibited in PBS treated group, indicating the extensive ROS in the RASFs. The ROS cleaning capability in the MHI and MAHI groups was stronger than that in PBS treated group but still inferior to the MAHI + Laser group, which suggested that the released MNPs and H_2_ could efficiently clean ROS either alone or synergistically. Moreover, the weakest green fluorescence intensity was shown in the MAHI + Laser group, suggesting that the increased temperature due to the PTT effects enhanced the antioxidant effectiveness of MAHI NGs. Excess inflammatory cytokines expression (IL‐6, IL‐1β, and TNF‐α) in RA lesion areas could accelerate the inflammatory response.^[^ ^38^ ^]^ Therefore, we investigated the variability of inflammatory cytokines after various treatments using the Enzyme‐Linked Immunosorbent Assay (ELISA) kits (Figure 3i–k). When MAHI NGs were incubated by the RASFs and then treated with laser irradiation, the expressions of IL‐6, IL‐1β, and TNF‐*α* were down‐regulated compared with other groups, indicating that the nanoagents could dramatically inhibit the inflammatory progress, consistent with the above DCFH‐DA results (Figure 3h). Overall, such hypoxic specific activated nanoagents had significantly advanced the achievement of synergistic treatment as follows: 1) The hypoxic microenvironment in RA specifically caused the degradation of MAHI NGs, leading to the release of loaded functional small molecules (MNPs, AB, and ICG) for achieving rapid and precise NIR‐II FL/PA dual‐modality imaging. 2) The MNPs not only serve as PA contrast agents but also facilitate efficient PTT to inhibit the proliferation of RASFs. 3) The H_2_ generated by the released AB could synergistically exert ROS scavenging and anti‐inflammation effects with MNPs, which could reduce lesion joint inflammation and oxidative damage successfully. 4) The increased temperature induced by PTT could expedite the release of H_2_ and enhance the antioxidant and anti‐inflammatory effects mediated by MNPs and H_2_.

**Figure 4:**
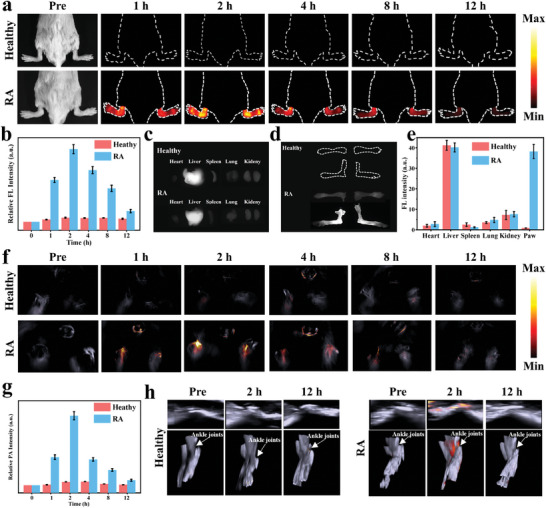
NIR‐II FL/PA imaging of MAHI NGs in vivo. a) NIR‐II FL imaging of healthy and CIA mice at different time points after receiving MAHI NGs under 808 nm excitation. b) Corresponding time‐course quantitative analysis of NIR‐II FL signal intensity post‐injection of MAHI NGs c,d) NIR‐II FL images for main organs and paws in vitro from two groups at 2 h post‐injection. e) NIR‐II FL signal intensity at different places in healthy mice and CIA mouse models after MAHI NGs injection. f) Time‐dependent PA images, g) quantification analysis, and h) 3D reconstruction images (pre, 2 h, and 12 h) of the joints in various groups after intravenous injection.

Given the incurable nature of RA traits in contemporary times, an accurate and timely diagnosis holds significant implications for effective RA treatment. Motivated by the excellent NIR‐II FL/PA imaging properties in vitro, the CIA mice models were constructed to assess the targeting ability and biological metabolism of MAHI NGs in vivo.^[^ ^39^ ^]^ As shown in **Figure** **4**a, we investigated the accumulation and clearance behavior of MAHI NGs in the inflamed joints following the collection of the NIR‐II FL imaging at various time intervals after injection. No visible NIR‐II FL signals were captured in the group of healthy mice after intravenous administration of MAHI NGs (20 mg kg^−1^). Interestingly, the fluorescence intensity in the inflamed joints of CIA mice grew specifically reached its peak at 2 h post‐injection MAHI NGs, which was matched with the quantified results (Figure 4b). The increased fluorescence intensity observed in inflamed joints could potentially be attributed to the “ELVIS” effect. Moreover, we harvested joints and primary organs in two groups at 2 h after intravenous administration of MAHI NGs to explore the biodistribution *ex vivo*. As shown in Figure 4c–e, while fluorescence signals were detected in the liver of mice at a certain intensity, the most prominent fluorescence signal was observed in the inflamed joints, further demonstrating the bioaccumulation of lesion joints and the distribution of the main organs.^[^ ^40^ ^]^

Subsequently, to further perfectly obtain comprehensive diagnostics, PA imaging could provide remarkably deep penetration and 3D images for adapting to complex tissue environments, thereby depicting more intuitive and accurate information about diseased tissues in RA joints. Compared to the blood, a six‐fold enhancement in PA intensity of MAHI NGs at 808 nm contributes to superior high‐contrast PA imaging (Figure S2, Supporting Information). According to Figure 4f, compared to the healthy mice, the PA signal in the inflamed joints of the CIA mice steadily increases with time, reaching a maximum level at 2 h post‐injection, similar to that of the NIR‐II FL imaging. Noticeably, NIR‐II PA images of the inflamed joints at 2 h displayed a significantly higher intensity compared with joints of healthy mice (Figure 4g). Additionally, the 3D reconstruction PA images of the joints provided high spatial resolution imaging for accurate RA diagnosis (Figure 4h). Taken together, NIR‐II FL and PA results demonstrated that MAHI NGs could efficiently target inflamed joints and reach an ideal time‐point at 2 h post‐injection for diagnosis and treatment of RA. Both NIR‐II FL and PA outcomes highlight the advantage of MAHI NGs in precisely targeting and accumulation in inflamed joints for RA diagnosis and therapy.

**Figure 5:**
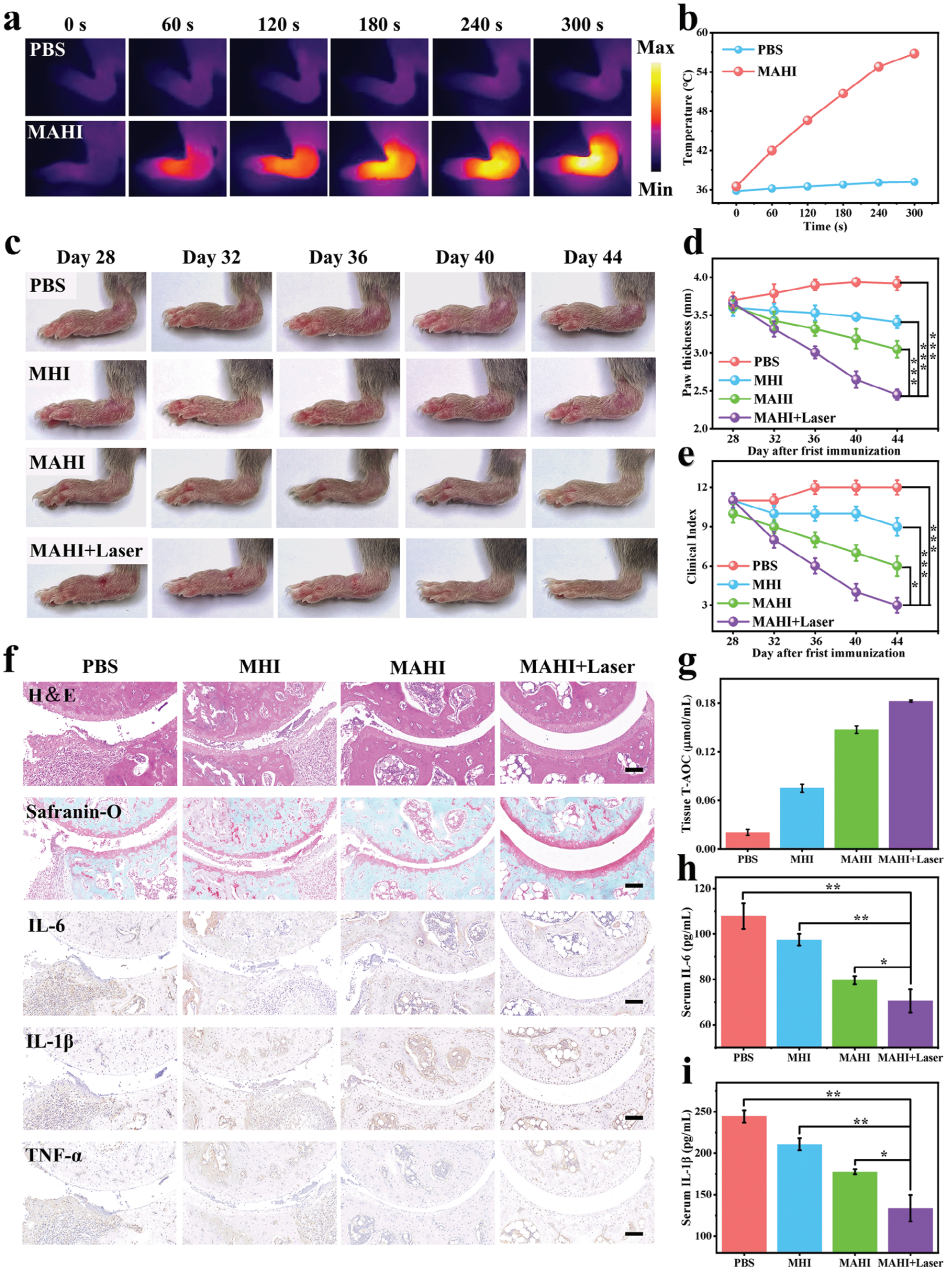
Therapy effect evaluation of MAHI NGs in vivo. a) Infrared thermography and b) temperature elevation curve of the ankle joint in the CIA mouse model injected with PBS or MAHI NGs during 5 min irradiation (808 nm, 1 W cm^−2^, 5 min). c) Representative images of diseased paws of CIA mouse models treated with PBS, MHI, MAHI, and MAHI + Laser during the treatment stage. d) Progression of paw thickness and e) clinical arthritis scores in the CIA mouse models with different formulations. f) Representative H&E, safranin‐O, and IHC staining of joint tissues taken from different groups. Scale bar: 100 µm. g) Antioxidant capability evaluation of inflammatory joints at the end of the experiment. Trolox was used as a control standard. h,i) Levels of proinflammatory cytokines IL‐6 and IL‐1*β* in serum. Mean ± SD, n = 3, ^*^ *p* < 0.05, ^**^ *p* < 0.01, ^***^ *p* < 0.001.

Considering the excellent absorption properties of MAHI NGs and the optimal therapy time‐point provided by dual‐model imaging, we captured temperature changes by infrared thermography imaging of the CIA mouse under 808 nm laser irradiation for 5 min to demonstrate the PTT efficacy of MAHI NGs in vivo. The CIA mouse models were intravenously injected with MAHI or PBS, and then the temperature change of the inflamed joints illuminated with a laser for 5 min was recorded. As depicted in **Figure** **5**a,b, the temperature gradually increased in the MAHI group, reaching a maximum of 56.8 °C after 5 min irradiation, while that of the PBS group only showed a slight rise. The rapidly increasing temperature indicated that the MAHI NGs can be efficiently accumulated in the inflamed joints to achieve an efficient PTT effect in vivo.

To assess the combined therapeutic effect in vivo, the CIA mice were randomly distributed into 4 different groups and treated with PBS, MHI, MAHI, and MAHI + Laser every 4 d for a total of 5 rounds. Paw thickness and clinical score were employed to access curative effect evaluation. Monitoring every 4 d in the treatment period, representative images of diseased paws were also recorded. As the disease progressed, the PBS‐treated CIA mouse showed severe paw swelling, erythema, and edema in the inflamed joints, consistent with the rapidly increasing clinical scores (Figure 5c–e). In contrast to the other groups, the CIA mouse treated with MAHI + Laser therapy showed the strongest suppression of RA and minimized the clinical scores. The changes in paw thickness were consistent with the clinical scores, in which the MAHI + Laser group showed a notable decline in paw thickness over time compared with the MHI and MAHI groups. This could be ascribed to the release of MNPs from the MAHI NGs, which can effectively ablate the RASFs via PTT. At the same time, the MNPs and the responsive release of H_2_ may exert distinctive antioxidant and anti‐inflammatory performances to alleviate RA development. Additionally, the amelioration of RA symptoms in both MHI and MAHI groups to varying degrees was presumably attributed to the antioxidant and anti‐inflammatory properties of the controlled release of MNPs and H_2_ from MAHI NGs. All of these outcomes confirmed that PTT combined with the superior antioxidant and anti‐inflammatory performance had a synergistic therapeutic effect on RA.

After treatment, we collected the joints of different groups for histological analysis (Figure 5f). The hematoxylin‐eosin (H&E) stained images in the PBS‐treated group significantly presented narrower joint cavities, evident synovial fibrosis, and bone erosion, which are typical characteristics of RA.^[^ ^41^ ^]^ In comparison, the MAHI + Laser group showed no significant synovitis and bone erosion, while partially alleviating inflammation of synovial inflammation was observed in both the MHI and MAHI groups. Furthermore, similar results were obtained in cartilage images stained with Safranin‐O, where the MAHI + Laser group showed much smoother articular facets than the other groups, indicating the success of MAHI + Laser in RA treatment with PTT, antioxidant, and anti‐inflammatory multi‐performances. In addition, the expression levels of inflammatory cytokines (IL‐6, IL‐1β, and TNF‐*α*) were assessed through immunohistochemical (IHC) staining. As shown in Figure 5f, the PBS‐treated group resulted in up‐regulated expression of these inflammatory cytokines, while the expression levels in the MHI and MAHI‐treated groups were less than the PBS group but still higher than the MAHI + Laser group. Compared with the above groups, the lowest expression of these inflammatory factors in the joint was observed in the MAHI + Laser group, indicating that the PTT, antioxidant, and anti‐inflammatory properties could synergistically treat RA and ameliorate the inflammatory microenvironment at lesion joints. Correspondingly, similar results were noted when the serum concentrations of IL‐6, IL‐1β, and TNF‐α were measured by ELISA kits in each group. The MAHI + Laser group exhibited much lower inflammatory factors than other groups (Figure 5h,i; Figure S3 Supporting Information). Moreover, we tested the total antioxidant properties (ABTS method, ordinate expressed as Total Antioxidant Capacity (T‐AOC)) from the joint tissue (Figure 5g). The MAHI + Laser group demonstrated the best antioxidant properties of all groups, corresponding to the effect at the cellular level. On the whole, the above results affirmed the synergy among the PTT, antioxidation, and anti‐inflammatory effects, demonstrating that the MAHI + Laser group with intrinsic multiple therapeutic functions offered a tremendous curative effect in RA.

## 3 Conclusion

In this work, we have successfully developed specifically activated nanoagents MAHI NGs to access the precise NIR‐II FL/PA imaging‐guided multi‐treatment paradigm for RA management. The precise theranostic procedure depended on the unique competency of the hypoxic‐responsive release functionalized components. In vitro, results revealed that the MAHI NGs could realize exceptional therapeutic efficacy in highly efficient PTT to ablate RASFs, mediate ROS scavenging, and downregulate inflammatory cytokines in the RA microenvironment. Furthermore, MAHI NGs, owing to superior NIR‐II FL/PA dual‐modal imaging properties, exhibited the exact time‐point of maximum accumulation in vivo in inflamed joints for theranostics of RA. Taken together, MAHI NGs were specifically activated by a hypoxic microenvironment to provide a novel multi‐treatment paradigm under efficient NIR‐II FL/PA imaging for precise theranostics of RA.

## 4 Experimental Section

Materials and experimental details are provided in the Supporting Information. All animal studies were performed in the Animal Experiment Center of Shanxi Medical University and the procedures involving experimental animals were in accordance with protocols approved by the Institutional Animal Care and Use Committee of the Animal Experiment Center of Shanxi Medical University (No. 2016LL141, Taiyuan, China).

## Conflict of Interest

The authors declare no conflict of interest.

## Author Contributions

L.C., M.Z., W.K., L.Y., and C.Z. contributed equally to this work.

## Supporting information

Supporting Information

## Data Availability

The data that support the findings of this study are available from the corresponding author upon reasonable request.
