# Supplementary material for: Endogenous Melanin and Hydrogen‐Based Specific Activated Theranostics Nanoagents: A Novel Multi‐Treatment Paradigm for Rheumatoid Arthritis
Source: Adv Sci (Weinh). 2024 Apr 26;11(25):2401046. doi: 10.1002/advs.202401046 (PMC11220692; doi:10.1002/advs.202401046)
Supplement: Supplementary file 1 — Supporting Information [file ADVS-11-2401046-s001.pdf]

ADVANCED SCIENCE
Open Access

Supporting Information

for *Adv. Sci.*, DOI 10.1002/advs.202401046

# Endogenous Melanin and Hydrogen-Based Specific Activated Theranostics Nanoagents: A Novel Multi-Treatment Paradigm for Rheumatoid Arthritis

*Lin Chen, Mingxin Zhao, Weiwei Kang, Lujie Yu, Chongqing Zhang, Shutong Wu, Xiaorui Song, Keqi Zhao, Pengmin Liu, Qin Liu, Rong Dai, Ziliang Zheng* and Ruiping Zhang**

# Endogenous Melanin and Hydrogen-Based Specific Activated Theranostics Nanoagents: A Novel Multi-Treatment Paradigm for Rheumatoid Arthritis

*Lin Chen* [a,b,1], *Mingxin Zhao* [a,1], *Weiwei Kang* [a,1], *Lujie Yu* [a,b,1], *Chongqing Zhang* [c,1], *Shutong Wu* [a], *Xiaorui Song* [a,b], *Keqi Zhao* [a,b], *Pengmin Liu* [d], *Qin Liu* [d], *Rong Dai* [a,c], *Ziliang Zheng* [a,b*], *and Ruiping Zhang* [a*]

[a] Department of Radiology, Fifth Hospital of Shanxi Medical University (Shanxi Provincial People's Hospital), Taiyuan, 030000, China

[b] Academy of Medical Sciences, Shanxi Medical University, Taiyuan, 030001, China

[c] Medical Imaging Department, Shanxi Province Cancer Hospital (Shanxi Hospital Affiliated to Cancer Hospital, Chinese Academy of Medical Sciences/Cancer Hospital Affiliated to Shanxi Medical University), Taiyuan, 030001, China

[d] Third Hospital of Shanxi Medical University, Shanxi Bethune Hospital, Shanxi Academy of Medical Sciences, Tongji Shanxi Hospital, Taiyuan 030032, China

## Experimental Section

### 1. Materials.

2-nitroimidazole (2-NI), Hyaluronic acid (HA, 10000 Da), Calcium carbonate ($CaCO_3$), amine-borane complex (AB), dimethyl formamide (DMF), dichloromethane ($CH_2Cl_2$), 1-(3-Dimethylaminopropyl)-3-ethylcarbodiimide hydrochloride (EDC·HCl, 98 %), and N-hydroxysuccinimide (NHS, 98 %) were acquired from Aladdin (Shanghai, China). Melanin (MNPs), indocyanine green (ICG), nicotinamide adenine dinucleotide (NADH), nitroreductase (NTR), Cell Counting Kit-8 (CCK-8), and AO/EB double fluorescence staining kit were purchased from Sigma-Aldrich. Total Antioxidant Capacity Assay Kit with a Rapid ABTS method (T-AOC Assay Kit) was purchased from Beyotime. Trifluoroacetic acid (TFA) was purchased from Beijing Bailingwei Technology (Beijing, China). 6-(Boc-amino) hexyl bromide and methylene blue (MB, 82 %) were purchased from Energy Chemical (Shanghai, China). The performed experiment utilized ultrapure water. All chemicals and reagents were of analytical grade and used without any further purification.

### 2. Characterization.

The morphology was observed using a JEOL-2100F transmission electron microscope (TEM). The UV-vis absorption was recorded on TU-1901 dual-beam UV-vis spectrophotometer (PerkinElmer). Zetasizer (Malvern Instruments Ltd.) was used to measure Dynamic light scattering (DLS) and zeta potential measurements. The photothermal temperature was acquired by an infrared camera (Fluke Ti400). The PA signal was recorded by a real-time multispectral optoacoustic tomographic (MSOT) imaging system. The NIR-II fluorescence emission spectra (900-1300 nm) was performed on a fluorescence spectrometer (Suzhou NIR-Optics Co., Ltd., China) equipped under a 808 nm laser.

### 3. Synthesis of amphiphilic NI-HA.

The synthesis of NI-HA is based on amide bond formation. Firstly, 6-(2-nitroimidazole) hexylamine (NI-$NH_2$) was prepared. 2-Nitroimidazole (0.34 g, 3 mmol) was dissolved in DMF (5mL) and $K_2CO_3$ (0.83 g, 6 mmol) was added. In this solution, 6-(Boc-amino) hexyl bromide (0.84 g, 3 mmol) dissolved in DMF was then dropped and the reaction took place for 12 h. After the reaction, the product dried the solvent, resuspending in water and extracted with ethyl acetate (15 mL) three times. Collected the organic layer and dried over anhydrous $Na_2SO_4$, then evaporated to afford the Boc-protected NI-$NH_2$ (NI-$NH_2$-Boc). Secondly, the yellow solids from the previous step were dissolved in $CH_2CH_2$ and added to the methanolic solution containing trifluoroacetic acid, stirring at room temperature for 4 h to deprotect the Boc group. The procedure was repeated three times to obtain the 2-nitroimidazole derivative (NI-$NH_2$) as yellow solids. Finally, the 2-nitroimidazole derivative (NI-$NH_2$) was conjugated to hyaluronic acid (HA). The HA (0.1 g, 0.25 mmol) was dissolved in water (5 mL) and stirred for 2 h, followed by adding EDC·HCl (0.192 g, 1 mmol) and NHS (0.115 g, 0.25 mmol), activated in ice bath for 0.5 h. After that, the 2-nitroimidazole derivative (NI-$NH_2$, 106 mg, 0.5 mmol) was slowly added and reacted for 24 h at room temperature. The product was transferred to the dialysis bag (MW = 8000 Da) after the reaction, dialyzed in methanol/water (V:V = 1:1) for 24 h, and in ultra-pure water for 48 h, and lyophilized to obtain nitroimidazole-grafted hyaluronic acid (NI-HA).

**4. Synthesis of MAHI NGs.**

MAHI NGs were prepared by self-assembly method. Firstly, NI-HA (100 mg) was dissolved in PBS (10 mL) and swollen for 12 h to obtain a polymer solution (10 mg/mL). Next, 0.5 mL of aminoborane (AB, 2 mg/mL), and 2 mL of melanin nanoparticles (MNPs, 5 mg/mL) were added sequentially with rapid stirring in an ice bath for 4 h. After ultrafiltration to remove the unencapsulated AB and MNPs, added 0.5 mL of ICG (2 mg/mL) in the above solution to load it on the surface of the nanoagents. Finally, MAHI NGs were centrifuged by ultrafiltration (MW = 10000 Da), and freeze-dried for use.

**5. Phototherapeutic effect of MAHI NGs *in vitro*.**

The photothermal heating curve was recorded to monitor the temperature change of MAHI NGs solutions with different concentrations for 5 min (1.0 W/cm$^2$) under the laser irradiation at 808 nm. The photostability of MAHI NGs was evaluated through five cycles between heating and cooling. The photothermal conversion was investigated by irradiating the MAHI NGs in aqueous solution (1.0 mL) with a laser (808 nm, 1.0 W/cm$^2$). The photothermal conversion efficiency of the MAHI NGs was calculated following a reported method. A standard formula was used as follows:

$$\eta = \frac{\mathrm{h}A(T_{max} - T_{sur}) - Q_{diss}}{I(1 - 10^{-A_\lambda})} = \frac{\mathrm{mc}(T_{max} - T_{sur}) - Q_{diss}}{\tau_s I(1 - 10^{-A_\lambda})}$$

"$T_{max}$" and "$T_{sur}$" are initial and the highest temperature of the MAHI NGs. "$Q_{diss}$" was measured by a Spectra-Physics power meter representing the heat dissipation. "*I*" represents the power of laser. "$A_\lambda$" is the absorbance at 808 nm. "*m*" represents the quality of MAHI NGs solution. "*c*" is the specific heat capacity of water. The value of "$\tau_s$" was calculated by the following formula.

$$\tau_s = -t/ln\theta$$

"*θ*" is the dimensionless driving force and "*t*" is the corresponding time.

**6. Hypoxia degradation and response test *in vitro*.**

To mimic tumor hypoxia in vitro, nitroreductase (10 μg/mL) and NADH (100 μM) were added to MAHI NGs solution (800 μg/mL) with PH 7.4. Transfer solution of MAHI NGs (2 mL) to a liquid flash vial and mix slowly at 37 °C. The mixed solutions were centrifuged by ultrafiltration (10 kDa) at different time points (0-6 h) to demonstrate degradation of the MAHI NGs nanoagents by detecting changes in absorption peak at 329 nm, DLS and TEM images.

**7. Detection of $H_2$ *in vitro*.**

The generation of hydrogen was tested with methylene blue (MB). The $H_2$ production rate of MAHI NGs under the condition of normal oxygen and hypoxia was evaluated respectively, and the effects of pH values and temperature on hydrogen production were detected.

**8. Evaluation of total antioxidant capability (T-AOC) in vitro and in vivo.**

The rapid 3-ethylbenzthiazoline-6-sulfonic acid (ABTS) method (Beyotime, China) was used to evaluate the total antioxidant capacity (T-AOC) of MAHI NGs. The ABTS radical cation ($ABTS^{+\cdot}$, blue/green) could be scavenged by substances with antioxidant properties, which caused changes in absorbance at 734 nm. Specifically, the $ABTS^{+\cdot}$ working solution was mixed with the solutions of different conditions, and the absorbance at 734 nm was detected with a microplate reader.

**9. Cell lines**

*9.1. Cell culture.*

Human umbilical vein endothelial cells (HUVECs) and rheumatoid arthfitissynovial fibroblasts (RASFs) were purchased from the Type Culture Collection of the Chinese Academy of Sciences (Shanghai, China). HUVECs and RASFs were cultured in DMEM high glucose medium that contain 1 % antibiotics (penicillin-streptomycin) and 10 %(v/v) fetal bovine serum (FBS) at 37 ℃ and 5 % $CO_2$ atmosphere.

*9.2. Cellular uptake test in vitro.*

RASFs were incubated with PPAC at 37 °C , 5%$CO_2$ cell culture incubator. Subsequently, we added MAHI NGs and incubated at different time, and than carefully washing with PBS buffer. RASFs were fixed with 4 % paraformaldehyde for observation of the NIR-II FL signals. Moreover, the PA imaging signals was observed after centrifugation of the RASF washed with PBS buffer.

*9.3. Cytotoxicity assay.*

The CCK-8 assay was used to investigate the *in vitro* cytotoxicity against HUVECs and RASFs. In brief, RASFs and HUVECs were seeded for 12 h at a density of $1.5×10^4$ cells/well in the 96-well plates to let cells attach. Subsequently, gradient concentrations of MAHI NGs were added cells were co-incubated for another 6 h. The CCK-8 working solution (10 %, V/V) was further incubated for 30 min under the same condition. Finally, the absorbance of each well at 450 nm was recorded. Finally, the living cells and the dead cells are stained by Trypan Blue Staining Solution.

*9.4. Intracellular ROS scavenging activity assay*.

First, RASFs ($1.0 × 10^4$ cells, DMEM high glucose medium, and 5% FBS) were inoculated into 96-well plates and incubated at 37 °C for 24 h to stimulate ROS production. Then, the cells were washed with PBS and incubated with different samples for 5 h. Intracellular ROS levels were obtained by fluorescence imaging at 488 nm excitation after 15 min labeling with DCFH-DA (10 μM in DMEM high glucose medium without FBS).

*9.5. ELISA kits.*

In the 6-well plates, the RASFs were planted with a density of $1×10^6$ cells per well. After gathering the culture material, ELISA kits were used to measure the amounts of interleukin-6 (IL-6), interleukin-1β (IL-1β), and tumor necrosis factor-α (TNF-α).

**10. Mice Models of Collagen Induced Arthritis (CIA).**

The DBA/1 mice were treated with intradermal injections of chicken type II collagen mixed the Freund's adjuvant (emulsion of two reagents at volume ratio = 1:1) at caudal root, followed by a booster immunization in 21 days using CII emulsified in incomplete Freund's Adjuvant. Mice were assessed for induction by measuring paw swelling with calipers or clinical scoring.

**11. Dual-modal imaging *in vivo*.**

To evaluate biodistribution *in vivo,* CIA mice were injected intravenously with MAHI NGs (20 mg/kg). They were anesthetized for NIR-II FL and PA imaging at 0, 1, 2, 4, 8, and 12 h post-injection. Main organs (hearts, livers, spleens, lungs, and kidneys) and paws were *ex vivo* NIR-II FL analyzed.

**12. Photothermal therapy *in vivo.***

CIA mice models were divided into two groups at random for various treatments: (1) PBS and (2) MAHI NGs. The whole ankle joint region was irradiated with 808 nm laser (1.0 $W/cm^2$, 5 min) after i.v. injection of the nanoagents. The average temperature in the ankle joint region was recorded by an infrared thermal imaging system.

**13. Effectiveness of treatment *in vivo*.**

We established the CIA mice models to test the therapeutic effect of the nanoagents *in vivo*. After 28 days, the mice were randomly divided into 4 groups (n = 3) for different treatments: (1) PBS group, (2) MHI group (100 μL, 4 mg/kg), (3) MAHI NG, (4) MAHI NGs+Laser (100 μL, 4 mg/kg, 1 $W/cm^2$, 5 min). Injections were made every 4 d for a total of 5 rounds. The hind paw thickness and arthritis score were monitored every other day.

**14. *In vivo* therapeutic effects evaluation.**

After treatment, the collected ankle joints were used for histological analysis using hematoxylin and eosin (H&E), safranin-O, and immunohistochemical staining. The mice's serum levels of TNF-α, IL-1β, and IL-6 were measured using the ELISA assay. Moreover, the periarticular tissues were collected for measurement of T-AOC level using a specific kit after treatment.

**15. Statistical analyses.**

All results were presented as the mean values ± standard deviation. The statistical significance was considered significance as a value of *P < 0.05, **P < 0.01, or ***P < 0.001, n.s. = not significant, respectively.

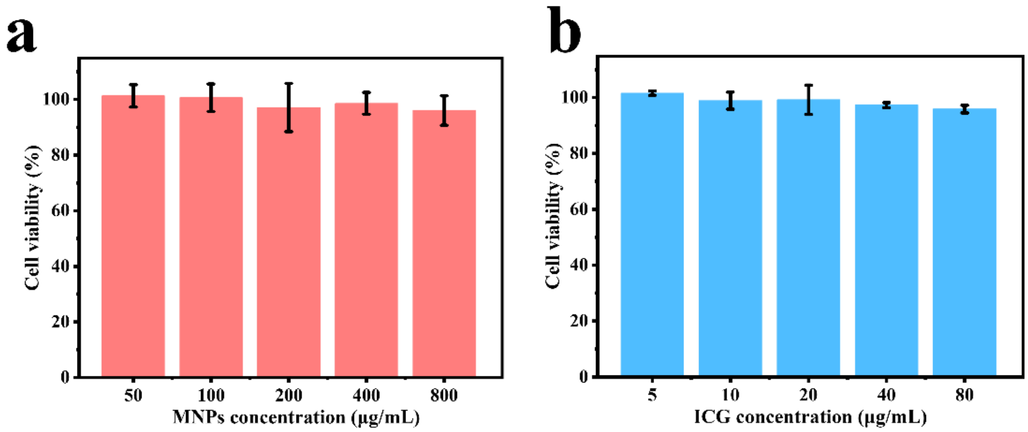


**Figure S1.** The Cell viability of HUVECs after incubation with MNPs (a) and ICG (b) in various concentrations.

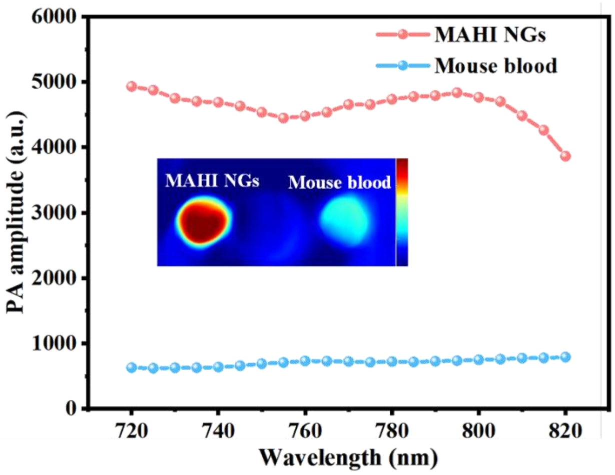


**Figure S2.** *In vitro* PA signal intensity of the MAHI NGs solution (800μg/mL) and mouse blood.

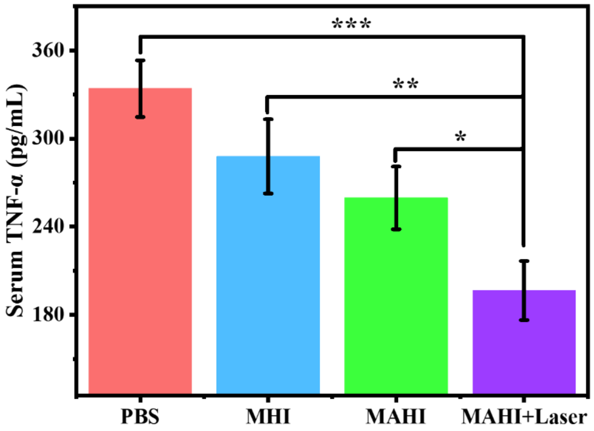


**Figure S3.** Levels of proinflammatory cytokines TNF-α in serum.
